# Single-nucleotide polymorphisms in a vancomycin-resistant *Staphylococcus aureus* strain based on whole-genome sequencing

**DOI:** 10.1007/s00203-020-01906-y

**Published:** 2020-06-13

**Authors:** Jung Wook Kim, Kwang Jun Lee

**Affiliations:** Division of Antimicrobial Resistance, Center for Infectious Diseases Research, National Institute of Health, Korea Centers for Disease Control and Prevention, Osong Health Technology Administration Complex, 187, Osongsaengmyeong 2-ro, Osong-eup, Heungdeok-gu, Cheongju-si, Republic of Korea

**Keywords:** *Staphylococcus aureus*, Vancomycin, VRSA

## Abstract

The emergence of vancomycin-resistant *Staphylococcus aureus* (VRSA) threatens global health. The mechanism of vancomycin resistance of VRSA without *vanA* gene acquisition was not fully elucidated. Therefore, we aimed to determine the mechanism of vancomycin resistance of VRSA besides that by *vanA* gene acquisition. In this study, we obtained vancomycin-resistant strains (V036-V64; MIC = 64 µg /ml) from susceptible strain (V036; MIC = 0.5 µg /ml) by exposure of vancomycin in vitro and examined the phenotypic characteristics and antibiotic susceptibility profiles of the resistant strain (V036-V64). To identify the genetic variations caused vancomycin resistance, we determined the complete genome sequences of V036 and V036-V64 and analyzed for single-nucleotide polymorphisms (SNPs) between two strains. Morphologically, V036-V64 had a twofold thicker cell wall compared with V036. Linezolid, rifampicin, and ceftaroline had similar MIC ranges against V036-V64 and V036, but V036-V64 showed lower susceptibilities to daptomycin and telavancin. We detected eight single-nucleotide polymorphisms differing between V036-V64 and V036: *rimM* (G16D), *ssaA2* (G128A), *rpsK* (P60R), *rpoB* (R917C), *walK* (T492R), d-alanyl-d-alanine carboxypeptidase (L307I), *vraT* (A152V), and chromosome segregation ATPase (T440I). This study demonstrates that, under selective pressure, by the accumulation of mutations in genes related to cell wall synthesis, vancomycin-susceptible *S. aureus* can develop thicker cell walls and, hence, develop high vancomycin resistance. Thus, we highlight a novel *vanA*-negative mechanism for VRSA emergence.

**Electronic supplementary material:**

The online version of this article (10.1007/s00203-020-01906-y) contains supplementary material, which is available to authorized users.

## Introduction

Methicillin-resistant *Staphylococcus aureus* (MRSA) is a major cause of nosocomial and community-associated infections throughout the world. MRSA can cause a variety of problems, from skin infections, sepsis, and pneumonia to bloodstream infections. Vancomycin is the main antimicrobial agent available to treat serious infections with MRSA. However, the intensive use of vancomycin over the years in healthcare premises has led to the development of strains with reduced vancomycin susceptibility.

*Staphylococcus aureus* strains with reduced vancomycin susceptibility are classified into two types depending on the level of vancomycin resistance. The first type is vancomycin-intermediate *S. aureus* (VISA), which was first reported in Japan in 1997 and against which vancomycin has a moderate minimum inhibitory concentration (MIC) (4–8 μg/ml) (Hiramatsu et al. 1997). VISA isolates do not carry imported foreign genetic elements; rather, the high vancomycin MIC values are related to the accumulation of mutations (Howden et al. 2014). The second type of strain is VRSA, first reported in the United States in 2002; the MIC of vancomycin is extremely high MIC (≥ 16 μg/ml) against VRSA (Sievert et al. 2008). Resistance in VRSA is conferred by the acquisition of the *vanA* gene by transposon Tn*1546* and plasmid transfer from vancomycin-resistant *Enterococcus faecalis* (Sievert et al. 2008). This *vanA*-type of VRSA is rare and geographically limited (Sievert et al. 2008; Tenover 2008). However, VISA strains are increasingly reported throughout the world (Howden et al. 2014).

It is noteworthy that *S. aureus* with reduced susceptibility to vancomycin is not restricted to humans. Recently, VRSA and VISA were reported to have been isolated from animals such as cattle, goats, and pigs (Adegoke et and Okoh 2014; Bhattacharyya et al. 2016; Kwok et al. 2013; Moreno et al. 2016).

In VISA, several gene mutations are known to contribute to the development of vancomycin resistance, including *ropB* (encoding a DNA-dependent RNA polymerase β-subunit), *walKR*, *vrsSR*, and *graSR* (encoding two-component regulatory systems) (Howden et al. 2010, 2014; Saito et al. 2014; Hu et al. 2016; Alam et al. 2014). However, the mechanisms underlying the progression of vancomycin resistance in VRSA by means other than *vanA* gene acquisition are not yet clear.

In this study, we aimed to examine the phenotypic characteristics and antibiotic susceptibility profiles of an induced vancomycin-resistant mutant and to identify the genetic variations causing vancomycin resistance.

## Materials and methods

### In vitro* selection of the induced vancomycin-resistant mutant*

A clinical isolate of vancomycin-susceptible *S. aureus* (VSSA) (V036; vancomycin MIC = 0.5) with sequence type (ST) 5 and staphylococcal cassette chromosome *mec* type II was used as the parental strain. Using V036 as the parental strain, a vancomycin-resistant mutant (V036-V64) was selected in vitro by serial passaging with progressively increasing concentrations of vancomycin. Briefly, overnight cultures of the vancomycin-susceptible strain in tryptic soy broth (TSB) were diluted 1:100 into fresh medium containing sub-inhibitory concentrations of vancomycin increased serially and incubated with shaking at 37 °C. To obtain a single vancomycin-resistant population, mutants grown in TSB containing 32 μg/ml vancomycin were selected again on tryptic soy agar (TSA) plates containing 64 μg/ml vancomycin. The vancomycin MICs of individual colonies picked at random were confirmed by the *E* test (bioMérieux, Marcy-I’Étoile, France). As a result, a stable mutant with a vancomycin MIC value of 64 μg/ml (V036-V64) was obtained. Chromosomal DNA digests (*Sma*I) of the strains V036-V64 and V036 showed identical pulsed-field gel electrophoretic profiles (data not shown). The mutant strain grown from a single colony was stored in 20% glycerol at − 80 °C.

### Antimicrobial susceptibility testing

Susceptibilities to ampicillin, azithromycin, clindamycin, cefoxitin, ciprofloxacin, daptomycin, erythromycin, gentamicin, linezolid, mupirocin, oxacillin, penicillin, rifampicin, quinupristin-dalfopristin, trimethoprim-sulfamethoxazole, teicoplanin, and vancomycin were tested using microdilution panels (MicroScan, Beckman Coulter Inc., Brea, CA, USA). Ceftaroline and telavancin MIC values were determined using the MIC test strip (Liofilchem, Roseto degli Abruzzi, Italy) and the Oxoid M.I.C.Evaluator™ (M.I.C.E™; Thermo Fisher Scientific, Basingstoke, England), respectively. The plates were incubated at 35 °C and read after 48 h. Population analysis was performed according to a reported method (Saito et al. 2014). Briefly, about 10^7^–10^8^ colony-forming units (CFU) of the overnight culture of each strain were inoculated on TSA plates containing various concentrations of vancomycin. The numbers of colonies formed after 48 h incubation at 35 °C were counted and plotted in a semilogarithmic graph.

### DNA extraction

Genomic DNA was extracted by using a Wizard genomic DNA preparation kit (Promega, Madison, WI, USA) according to the manufacturer’s protocol for bacterial cells. Lysostaphin was added at a final concentration of 30 µg/ml in lysis buffer, followed by incubation for 1 h at 37 °C. DNA quality and quantity were evaluated by agarose gel electrophoresis and using a Nanodrop (Thermo Fisher Scientific) spectrophotometer, respectively.

### Whole-genome sequencing and annotation

Whole-genome sequencing (WGS) for all strains was performed using an Illumina MiSeq system (Illumina Inc., San Diego, CA, USA). The sequencing library was prepared with the TruSeq DNA LT Sample Prep kit (Illumina) according to the manufacturer’s instructions. Sequencing was performed with 300 bp paired-end reads to a coverage of over 200X. The generated paired-end sequencing reads were assembled using SPAdes 3.10.0 (Bankevich et al. 2012). Gene prediction was performed using Glimmer 3 (Delcher et al. 2002), and annotation was conducted by homology search against the Clusters of Orthologous Groups (COG) and SEED databases (Disz et al. 2010; Tatusov et al. 1997). For validation, the assembled sequences were compared with the reference genomes of two MRSA strains, N315 and Mu50.

### Single-nucleotide polymorphism calling

MUMmer version 3.23 (Delcher et al. 2002) was used to align each of the de novo assemblies to a reference genome. Single-nucleotide polymorphisms (SNPs) were then called from the resulting alignments with a MUMmer application named show-snps (with options-Clr).

### Nucleotide sequence accession numbers

The complete nucleotide sequences of strains V036 and V036-V64 were submitted to GenBank under accession numbers PJIG00000000 and PJIH00000000, respectively.

## Results

### Characterization of the V036-V64 strain

The colony size of V036-V64 (1 mm) on the plate was smaller than that of V036 (3 mm). The colonies of V036-V64 on the plate were homogeneous in size, indicating the genetic stability of the strain (Fig. S1). Thickened cell wall peptidoglycan layers are the mechanistic features of the VISA phenotype. To evaluate the change in cell wall thickness in V036-V64, transmission electron microscopy was performed, which revealed that V036-V64 (34.96 ± 7.73 nm) had a thicker cell wall compared to V036 (17.71 ± 1.97 nm) (Fig. S1). Furthermore, the V036-V64 strain grew extremely slowly (Fig. S2). The average ± standard deviation DTs of the V036 and V036-V64 strains were 32.3 ± 3.9 min and 79.1 ± 3.0 min, respectively. Next, the V036-V64 strain had higher vancomycin resistance than that of the parent V036 strain; V036-V64 exhibited about 10^6^ CFU at a vancomycin concentration of 64 μg/ml (Fig. S2). Because the growth rate of the V036-V64 strain was too slow for us to determine the MIC after 24 h, the MICs of both V036 and V036-V64 strains were assessed after 48 h. The MICs of most of the tested antibiotics, including beta-lactams, macrolides, lincosamides, and fluoroquinolones, were unchanged in V036-V64 compared to V036 strain (Table 1). Meanwhile, the MICs of teicoplanin (glycopeptide) and telavancin (lipoglycopeptide) against V036-V64 were 16-fold and 23-fold higher, respectively, than those against V036. Daptomycin (lipopeptide) also had a higher MIC against the V036-V64 strain.

**Table 1:** Antibiotic susceptibilities of vancomycin-susceptible (V036) and vancomycin-resistant (V036-V64) strains

Strains	Minimum inhibitory concentration (μg/ml)																	
	AMP	AZI	CLI	CEF	CIP	CPT	DAP	ERY	GEN	LZD	MUP	OXA	RIF	QD	T/S	TLV	TEC	VAN
V036	> 8	> 4	> 2	> 4	> 2	≤ 1	0.25	> 4	> 8	≤ 1	≤ 4	≥ 256	0.004	≤ 1	0.5	0.047	0.5	0.5
V036-V64	8	> 4	> 2	> 4	> 2	≤ 1	4	> 4	> 8	≤ 1	≤ 4	≥ 256	0.004	≤ 1	0.5	0.25	3	64

*AMP* ampicillin, *AZI* azithromycin, *CLI* clindamycin, *CEF* cefoxitin, *CIP* ciprofloxacin, *CPT* ceftaroline, *DAP* daptomycin, *ERY* erythromycin, *GEN* gentamicin, *LZD* linezolid, *MUP* mupirocin, *OXA* oxacillin, *PEN* penicillin, *RIF* rifampicin, *QD* quinupristin/dalfopristin, *T/S* trimethoprim/sulfamethoxazole, *TLV* telavancin, *TEC* teicoplanin, *VAN* vancomycin

### Genetic changes in the VRSA phenotype compared with the VSSA strain V036

We determined the complete nucleotide sequences of the strains V036 and V036-V64 as 2,770,222 bp and 2,770,222 bp, respectively. The 2800 genes (2274 coding sequences, two pseudogenes, 16S rRNAs, and 58 tRNAs) were annotated with the MiGAP program. The GC content of V036 was 32.8%. This gene number and GC percentage were very similar to those of the N315 sequence (2787 genes and GC content of 32.8%).

To investigate the genetic changes in V036-V64 compared to V036, we analyzed the SNPs between the two strains. Remarkably, only eight SNPs were detected between V036 and V036-V64 (Table 2). The eight detected SNPs were non-synonymous substitutions, i.e., G16D, G128A, P60R, R917C, T492R, L307I, A152V, and T440I, located in *rimM*, *ssaA2*, *rpsK*, *rpoB*, *walK*, *pbp4*, *vraT*, and chromosome segregation ATPase genes, respectively.

**Table 2:** List of SNPs identified in V036-V64

No.	Locus ID	Gene	Protein product	Nucleotide change	Amino acid change
1	SAV036_00730	*rim*	16S rRNA-processing protein RimM	G → A	16, G → D
2	SAV036_00882	*ssA2*	Staphylococcal secretory antigen ssaA2	C → G	128, G → A
3	SAV036_00954	*rpsK*	30S ribosomal protein S11 rpsK	C → G	60, P → R
4	SAV036_01208	*rpoB*	DNA-directed RNA polymerase beta subunit RpoB	C → T	917, R → C
5	SAV036_01614	*walk*	Two-component histidine kinase sensor WalK	C → G	492, T → R
6	SAV036_02325		d-Alanyl-d-alanine carboxypeptidase (penicillin-binding protein 5/6)	Insertion (C)	307, L → I
7	SAV036_02688	*vraT*	Two-component histidine kinase sensor VraT	G → A	152, A → V
8	SAV036_02818		Chromosome segregation ATPase	A → T	440, T → I

## Discussion

The prevalence of superbugs, such as MRSA and even VRSA, is increasing rapidly both in hospitals and in the community. To date, 12 VRSA isolates have been reported in the United States, all of which belong to a phylogenetic lineage known as clonal complex 5 (Sievert et al. 2008). Although the majority of reports have been limited to the United States, with a few from other parts of the world (e.g., Iran) (Shekarabi et al. 2017; Thati et al. 2011; Tiwari and Sen 2006), the recent detection of a relatively high frequency of VRSA indicates the possibility of the dissemination of VRSA strains in various parts of the world. Furthermore, *vanA*-negative strains among these VRSA strains have also been reported. However, vancomycin resistance mechanisms of VRSA other than *vanA* gene acquisition, especially the underlying genetic changes, are still not understood fully.

In this study, to gain enhanced understanding of the genetic characteristics of the vancomycin resistance of VRSA, we obtained a vancomycin-resistant strain with the same genetic background as that of the susceptible strain ST5 by vancomycin selection in vitro. Next, we performed WGS of VSSA and its isogenic mutant VRSA strain and analyzed the genetic variations between the two strains. Herein, we identified eight non-synonymous mutations in V036-V64, including G16D in *rimM*, G128A in *ssaA2*, P60R in *rpsK*, R917C in *rpoB*, T492R in *walK*, L307I in SAV036_02325 (encoding d-alanyl-d-alanine carboxypeptidase), A152V in *vraT*, and T440I in SAV036_02818 (encoding chromosome segregation ATPase). RimM, a ribosomal maturation factor, and the ribosomal protein S2 are important proteins involved in protein synthesis. More specifically, RimM is an accessory factor required for 30S maturation and assembly in *Escherichia coli* (Lӧvgren et al. 2004), and the deletion of *rimM* has been known to decrease the growth rate and reduce translational efficiency at 37 °C (Bylund et al. 2001). *rpsK* encodes the 30S ribosomal protein S11. Mutation of this gene showed cell separation, swimming defects, and biofilm formation acceleration in *Bacillus subtilis* (Takada et al. 2014). The mutation of these two genes and SAV036_02818 (encoding chromosome segregation ATPase) might affect the growth rate of the V036-V64 strain via decreased chromosome segregation and cell separation in the V036-V64 strain. *S. aureus* SsaA homologs have been reported to share a common cysteine, histidine-dependent aminohydrolase/peptidase-amidase domain, and amino-terminal signal sequences, indicating that they are likely to be exported and/or targeted to the cell wall or membrane. SsaA is involved in cell wall metabolism and is regulated by the *walKR* two-component system in *S. aureus* (Dubrac et al. 2007; Hu et al. 2016). In addition, we found several mutated genes that were previously reported in VISA, such as *vraT*, *walK*, and *rpoB* involved in transcriptional regulation (Bankevich et al. 2012; Boyle-Vavra et al. 2013). VraTSR is a three-component system that regulates cell wall stress stimulon and the VraT is a negative regulator of VraSR (Hu et al. 2016). The VraT-Y220C mutation mediates the vancomycin-resistant phenotype in the ST8-USA300 strain of the VISA isogenic series of SG-S, SG-R, and SG-rev (Gardete et al. 2012). As the only known essential two-component regulatory system for the viability of *S. aureus*, the WalKR system connects cell wall biosynthesis with cell division (Howden et al. 2011). It has been best studied in *B. subtilis*, *S. aureus*, and *Streptococcus pneumonia*e where cell wall metabolism genes dominate the predicted regulon of *walR* (Howden et al. 2011). The *walKR* mutation is the most frequent in VISA strains (Howden et al. 2011). Several mutations within the WalK, including G223D, V268F, N382S, A567D, and T595I, have been reported (Howden et al. 2011), and the positions of mutations are not limited to specific domain or region. In this study, the R492C mutation of *walK* in V036-V64 strain was detected, which was different from those previously described in VISA. This suggests that the novel mutation sites were important for the development of resistance to vancomycin beyond VISA levels.

Recently, Ishii et al. reported the experimental development of vancomycin-resistant strains (Ishii et al. 2015). They generated a highly vancomycin-resistant *S. aureus* mutants by exposure with ethyl methanesulfonate. Eleven genes, including *graS*, *ausA*, *rpoB*, *rpoC*, *yloV*, *ebhA*, SA1593, *perR*, *sdrC*, *terC*, and SA1584, were mutated in two high-MIC (vancomycin MIC = 32 μg/ml) strains of ST5. Among them, only one gene (*ropB*) was consistent with our results. However, the mutation sites in *ropB* gene were different each other; L1083F in VR3, M978I in VR7 (Ishii et al. 2015), and R917C in our study. Two mutation sites of M978I and R917C were located in the RNA polymerase Rpb2 domain 6 (position 674-1069). This domain contains the important structural motifs, switch 3 and the flap loop, and binds an active site metal. This domain is also involved in binding to Rpb1 and Rpb3 (Cramer et al. 2001). The mutation site of H481Y previously reported in VISA (Alam et al. 2014) is located in domain 3 (position 468-536). This domain is known as the fork domain and is proximal to catalytic sites (Cramer et al. 2001). These suggest that the development pathways for obtaining high vancomycin resistance beyond intermediate resistance could vary. Above all, mutations detected in vancomycin-resistant strains need to be verified experimentally.

Although the genomic information from whole-genome sequencing approach are powerful hypothesis generator, genetic manipulation experiments are necessary to confirm functions of individual genes. In this study, a prospective trial was performed using two-step allelic exchange method described previously (Bae and Schneewind 2006), which requires cloning of the upstream and downstream sequences of a locus of interest into a pKOR1 plasmids. However, eventually failed to obtain knockout mutant strains.

*Staphylococcus aureus* V036-V64 strain was difficult to work due to two barriers in genetic manipulation experiments. First, a major barrier to the genetic manipulation of *Staphyococci* is the inability to transform plasmid DNA into the majority of clinical isolates due to a strong restriction-modification (RM) barrier. We have observed that *S. aureus* V036-V64 strain had type I RM system encoded by a *sau1hsdR* gene on the chromosome and by two copies of *sau1hsdM* and *sau1hsdS* on genomic islands GIα and GIβ. Most strains of *S. aureus* possess a strong restriction barrier that hinders exchange of DNA. These RM systems present in *S. aureus* have limited functional genomic analysis to a small subset of strains that are amenable to genetic manipulation. The RM systems are classified into type I, II, and IV RM systems. (Monk and Foster 2012). The most widely distributed RM systems present and functional in majority of strains of *S. aureus* are the SauI type I RM system (Waldron and Lindsay 2006). Type I RM systems comprise genes that encode a host specificity of DNA (hsd) specificity (S) protein, a modification (M) protein, and restriction (R) endonuclease (Murray 2000). Type I RM systems protect self-DNA from cleavage by catalyzing methylation of hemi-methylated residues within a target sequence whilst also recognizing foreign unmethylated DNA at the same target sequence and cleaving DNA at a site distant to the target sequence (Waldron and Lindsay 2006). The type II RM system consists of DNA methylases and restriction endonuclease enzymes. There are at least 11 subclasses of type II RM enzymes, all of which recognize specific DNA sequences and digest the DNA at either specific points or a pre-determined distance away from the recognition site to yield restricted fragments. There are two type II RM enzymes in *S. aureus*, SauAI, which recognizes the double stranded GATC site and Sau961, which recognizes the DNA sequence GGNCC (Jones et al. 2015). However, these restriction endonuclease enzymes are only found in a small number of *S. aureus* strains (Stobberingh et al. 1977). The type IV systems is the simplest form of restriction system with a single protein able to detect the methylation status. The type IV restriction enzyme that recognizes cytosine methylated DNA has been shown to be the major barrier to transfer of plasmid DNA from *Escherichia coli* into *Staphylococci*.

Second, thickened cell wall peptidoglycan layers of the V036-V64 strain lowered the probability of electroporation. Optimization of conditions for generating competent cells for electroporation will be required to increase the frequency of transformation. However, the whole process of generating of mutants is a laborious and time-consuming process. To improve progress towards a more comprehensive understanding of *S. aureus* biology, the development of simple genetic manipulation methods for all *S. aureus* strains is necessary.

Telavancin is a semisynthetic lipoglycopeptide derivative of vancomycin. Telavancin has a glycopeptide core that binds with high affinity to the acyl-d-alanyl-d-alanine terminus of cell wall precursors through a network of hydrogen bonds and hydrophobic packing interactions. Thus, telavancin, like vancomycin, inhibits peptidoglycan by binding to late-stage peptidoglycan precursors. In this study, telavancin was observed to have a 23-fold higher MIC (0.064–1.5 μg/ml) against the V036-V64 strain than that against the V036 strain. This result is consistent with the previous reports, wherein telavancin showed high MIC against VISA isolates (Draghi et al. 2008; Karlowsky et al. 2015). Daptomycin interferes with bacterial cell membrane function (Howden et al. 2011). The MICs of this bacterial cell membrane-targeting lipopeptide antibiotic were ≤ 1 μg/ml and 4 μg/ml against the V036 and V036-V64 strains, respectively. This result is consistent with the previous report, wherein single-nucleotide substitutions within either *walK* or *walR* lead to co-resistance to vancomycin and daptomycin (Howden et al. 2011). These results suggest that the thickening of the cell wall decreases the interaction of cell wall-targeting antibiotics with peptidoglycan and its access to the cell membrane.

In conclusion, this study demonstrated that prolonged vancomycin exposure leads to the development of vancomycin resistance in MRSA strains. Compared with the vancomycin-susceptible parental strain, eight SNPs were detected in a high-level vancomycin-resistant strain. These genes are related with cell wall metabolism and growth defects. Description of new SNPs in the core genome, which can be linked with *S. aureus* highly resistance to vancomycin will allow development of better and more rapid diagnostic methods for identifying of drug resistance. It can also provide insights into the genetic basis of acquiring vancomycin resistance (apart from *vanA* gene acquisition) which are crucial for understanding and managing the emergence of new VRSA strains. In the road ahead, the relationships between the above gene mutations and resistance need to be analyzed to clarify the evolutionary pathways underpinning the development of high resistance to vancomycin.

## Electronic supplementary material

Below is the link to the electronic supplementary material.

Supplementary file 1 (PDF 146 kb)

Supplementary Figure S1 (TIF 790 kb)

Supplementary Figure S2 (TIF 296 kb)
