# Supplementary material for: Single-nucleotide polymorphisms in a vancomycin-resistant Staphylococcus aureus strain based on whole-genome sequencing
Source: Arch Microbiol. 2020 Jun 13;202(8):2255–61. doi: 10.1007/s00203-020-01906-y (PMC7455577; doi:10.1007/s00203-020-01906-y)
Supplement: Supplementary file 1 — (PDF 146 kb) [file 203_2020_1906_MOESM1_ESM.pdf]

Supplementary materials

Materials and methods

Growth curve

Overnight cultures of strains in TSB were diluted 1:100 in fresh medium and incubated at 37℃ with shaking at 200 rpm. The optical density at 600 nm was measured every 30 min by using a spectrophotometer. The doubling time (DT) was calculated by the slope of the linear curve obtained from the semilogarithmic graph of the growth curve as follows: $DT = [(t2 - t1) \times \log 2]/(\log OD_{600} \text{ at } t2 - \log OD_{600} \text{ at } t1)$, where $t2$ and $t1$ are the times at the end and start of the logarithmic growth phase, respectively (Laurent et al. 2001). All growth experiments were performed in triplicate.

Transmission electron microscopy

All the strains were inoculated on TSB agar at 35℃ for 24 h and then fixed with 2% paraformaldehyde and 2% glutaraldehyde in 0.05 M sodium cacodylate buffer (pH 7.2) for 18–24 h. Samples were post-fixed for 2 h at room temperature with 1% osmium tetroxide in 0.05 M sodium cacodylate buffer (pH 7.2). The cells were then washed, dehydrated with ethanol, and embedded. Ultrathin sections (60–70 nm) were stained with uranyl acetate and lead citrate and observed using a JEM 1010 transmission electron microscope (JEOL, Tokyo, Japan) operating at 80 kV. Morphometric evaluation of the cell wall was performed using images at a final magnification of 100,000X. At least 30 cells from each strain with nearly equatorial cut surfaces were measured, and the results were expressed as mean ± standard deviation. The statistical significance of increases in the cell wall thickness was evaluated by Student’s *t*-test ($p < 0.001$).

Reference

Laurent F, Lelièvre H, Cornu M, Vandenesch F, Carret G, Etienne J, Flandrois J-P (2001) Fitness and competitive growth advantage of new gentamicin-susceptible MRSA clones spreading in French hospitals. J Antimicrob Chemother 47:277-283 doi: 10.1093/jac/47.3.277

Figure legends

Figure S1. Morphological appearance of V036 and V036-V64 colonies on TSA after incubation for 24 h (upper panel) and 48 h (bottom panel) at 35℃ (a). Transmission electron microscopy images of V036 (left) and V036-V64 (right) strains (b). Magnification: 100,000×. Data are expressed as mean ± SD values for cell wall thickness (nm).

Figure S2. Growth curve of the V036 and V036-V64 strains (a). Vancomycin population analysis profile of V036 and V036-V64 (b).
