# Supplementary figures and images for: Single-nucleotide polymorphisms in a vancomycin-resistant Staphylococcus aureus strain based on whole-genome sequencing
Source: Arch Microbiol. 2020 Jun 13;202(8):2255–61. doi: 10.1007/s00203-020-01906-y (PMC7455577; doi:10.1007/s00203-020-01906-y)

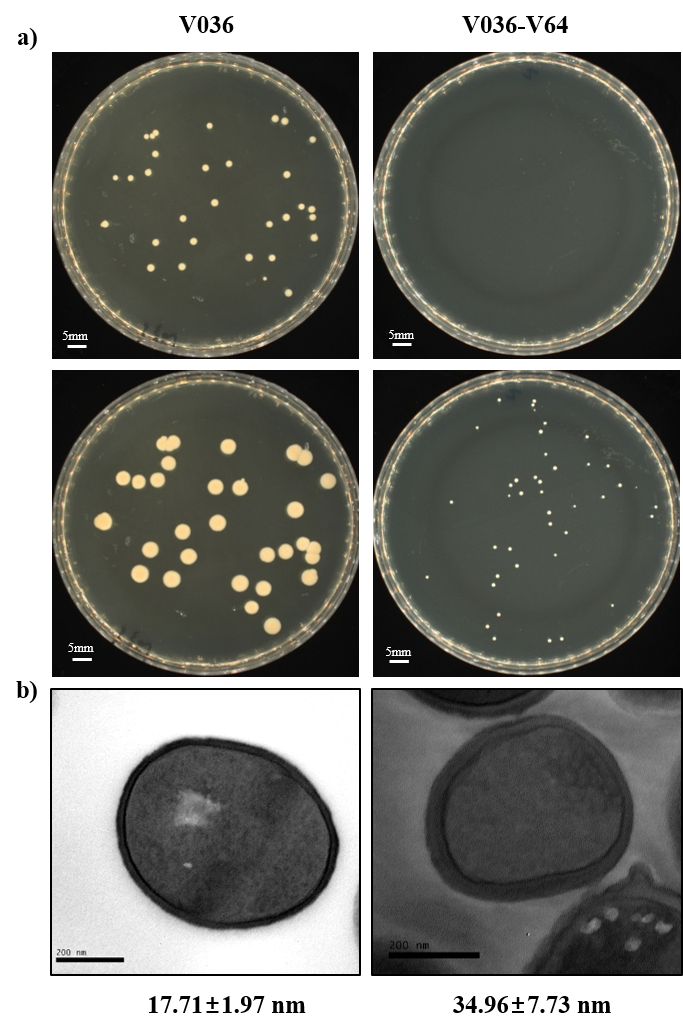

Supplement: Supplementary file 2 — Supplementary Figure S1 (TIF 790 kb) [file 203_2020_1906_MOESM2_ESM.tif]

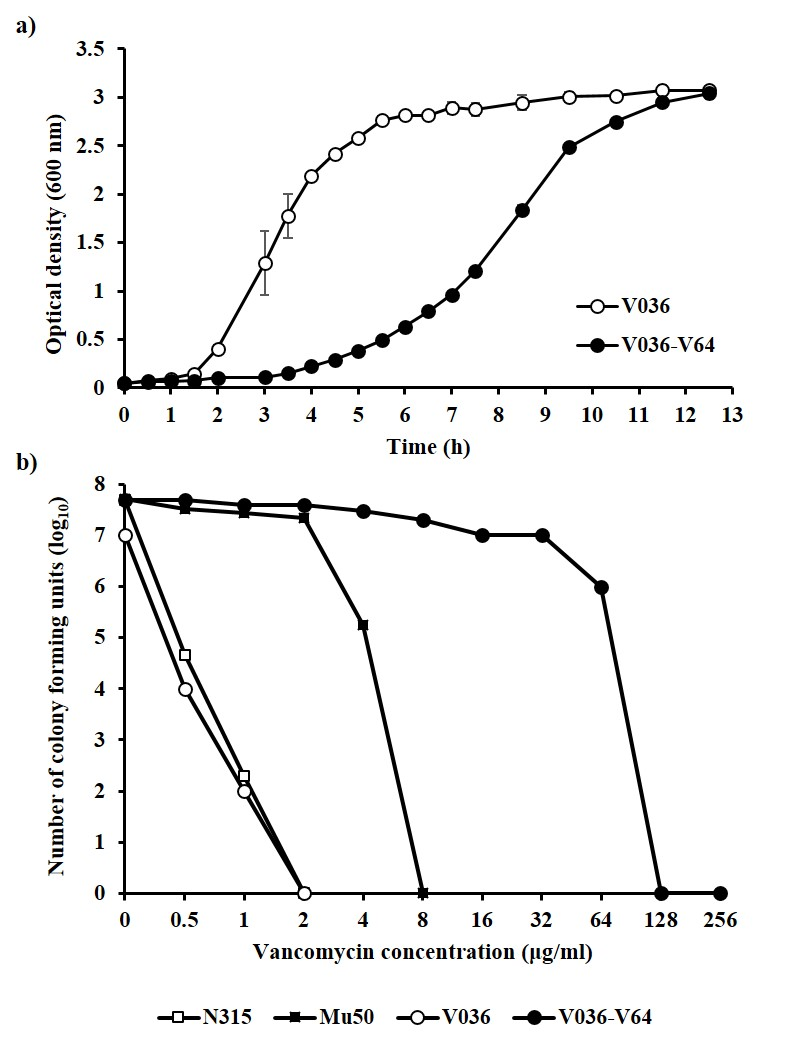

Supplement: Supplementary file 3 — Supplementary Figure S2 (TIF 296 kb) [file 203_2020_1906_MOESM3_ESM.tif]
